# A RAD-Based Genetic Map for Anchoring Scaffold Sequences and Identifying QTLs in Bitter Gourd (*Momordica charantia*)

**DOI:** 10.3389/fpls.2018.00477

**Published:** 2018-04-12

**Authors:** Junjie Cui, Shaobo Luo, Yu Niu, Rukui Huang, Qingfang Wen, Jianwen Su, Nansheng Miao, Weiming He, Zhensheng Dong, Jiaowen Cheng, Kailin Hu

**Affiliations:** ^1^College of Horticulture, South China Agricultural University, Guangzhou, China; ^2^Key Laboratory of Biology and Germplasm Enhancement of Horticultural Crops in South China, Ministry of Agriculture, College of Horticulture, South China Agricultural University, Guangzhou, China; ^3^Vegetable Research Institute, Guangdong Academy of Agricultural Sciences, Guangzhou, China; ^4^Tropical Crops Genetic Resources Institute, Chinese Academy of Tropical Agricultural Sciences, Danzhou, China; ^5^Vegetable Research Institute, Guangxi Academy of Agricultural Sciences, Nanning, China; ^6^Crops Research Institute, Fujian Academy of Agricultural Sciences, Fuzhou, China; ^7^Hunan Vegetable Research Institute, Changsha, China; ^8^Institute of Vegetables and Flowers, Jiangxi Academy of Agricultural Sciences, Nanchang, China; ^9^Beijing Genomics Institute, Shenzhen, China

**Keywords:** bitter gourd, restriction site associated DNA (RAD), genetic map, genome assembly, genetic location

## Abstract

Genetic mapping is a basic tool necessary for anchoring assembled scaffold sequences and for identifying QTLs controlling important traits. Though bitter gourd (*Momordica charantia*) is both consumed and used as a medicinal, research on its genomics and genetic mapping is severely limited. Here, we report the construction of a restriction site associated DNA (RAD)-based genetic map for bitter gourd using an F_2_ mapping population comprising 423 individuals derived from two cultivated inbred lines, the gynoecious line ‘K44’ and the monoecious line ‘Dali-11.’ This map comprised 1,009 SNP markers and spanned a total genetic distance of 2,203.95 cM across the 11 linkage groups. It anchored a total of 113 assembled scaffolds that covered about 251.32 Mb (85.48%) of the 294.01 Mb assembled genome. In addition, three horticulturally important traits including sex expression, fruit epidermal structure, and immature fruit color were evaluated using a combination of qualitative and quantitative data. As a result, we identified three QTL/gene loci responsible for these traits in three environments. The QTL/gene *gy*/*fffn*/*ffn*, controlling sex expression involved in gynoecy, first female flower node, and female flower number was detected in the reported region. Particularly, two QTLs/genes, *Fwa/Wr* and *w*, were found to be responsible for fruit epidermal structure and white immature fruit color, respectively. This RAD-based genetic map promotes the assembly of the bitter gourd genome and the identified genetic loci will accelerate the cloning of relevant genes in the future.

## Introduction

Bitter gourd (*Momordica charantia*; 2*n* = 2*x* = 22) is a tropical and subtropical vine in the family Cucurbitaceae, which is widely cultivated in Asia, Africa, and the Caribbean for its edible and medicinal fruit (Grover and Yadav, 2004; Marr et al., 2004; Van Wyk, 2015). Bitter gourd fruit provides a good source of phytonutrients like carbohydrates, minerals, and vitamins (Behera et al., 2010). The pharmacological composition and properties of bitter gourd have also been widely investigated (Tan et al., 2016). Although bitter gourd is consumed and cultivated, it remains an underutilized crop and scant efforts have been made for molecular improvement.

The construction of a genetic map is a common approach to detect QTLs and conduct gene mapping. The first genetic map of bitter gourd was constructed based on amplified fragment length polymorphism (AFLP) markers (Kole et al., 2012). A year later, the second genetic map was constructed, and it consisted of combined markers including simple sequence repeats (SSR), AFLP, and sequence-related amplified polymorphism (SRAP) markers (Wang and Xiang, 2013). Recently, two RAD-based genetic maps were constructed using two F_2_ populations derived from the same parents (Matsumura et al., 2014; Urasaki et al., 2017), allowing for preliminary attempts at next-generation sequencing (NGS). Urasaki et al. (2017) reported a bitter gourd reference (OHB3-1 reference) and anchored 255 scaffolds to the genetic map. RAD markers are short fragments of DNA adjacent to each instance of a particular restriction enzyme recognition site (Baird et al., 2008). Combined with NGS, RAD-seq provides an inexpensive platform that allows high-density SNP discovery and genotyping in large populations (Davey et al., 2011).

In order to conduct marker-assisted selection (MAS) in bitter gourd breeding, it is necessary to target genetic loci underlying important horticultural traits. Similar to cucumber and melon, bitter gourd shows different sexual morphs. Gynoecy has potential applications in heterosis breeding and hybrid seed production. The gene that controls gynoecy (*gy*) was reported as a single recessive gene (Ram et al., 2006; Behera et al., 2009; Matsumura et al., 2014). An identified SNP marker linked to this gene has been mapped at a distance of 5.46 cM (Matsumura et al., 2014). In addition, bitter gourd fruits show extensive variation in epidermal color and structure, which directly effects exterior quality. Immature bitter gourd fruits have a broad color spectrum, ranging from white to dark green (Cheng et al., 2012). Several studies have shown that the green fruit color is monogenically dominant over the white fruit color (Srivastava and Nath, 1972; Hu et al., 2002; Dalamu et al., 2012). However, a more recent study indicated that this trait is controlled by quantitative genes (Huang and Hsieh, 2017). Bitter gourd fruit has either a smooth or a distinct warty exterior, and has broken or continuous ridges. The warty exterior is monogenically dominant over the smooth (Kole et al., 2012); however, research devoted to identifying genetic loci responsible for these important traits is limited.

As part of our ongoing efforts to sequence the bitter gourd genome, an efficient genetic map is instrumental for anchoring and orienting the assembled scaffolds. Here, we report a RAD-based linkage map of bitter gourd using an F_2_ mapping population with 423 individuals derived from two cultivated inbred lines, the gynoecious line ‘K44’ and the monoecious line ‘Dali-11.’ Another major goal of the study was to map genetic loci underlying horticulturally important traits such as sex expression, fruit epidermal structure, and immature fruit color. To reach this objective, we carried out an elaborate investigation of phenotypic characters in three environments. The results of this study will be beneficial to research attempting to develop linked molecular markers in MAS breeding and to clone these genes in the future.

## Materials and Methods

### Mapping Population and Phenotype Evaluation

**FIGURE 1:**
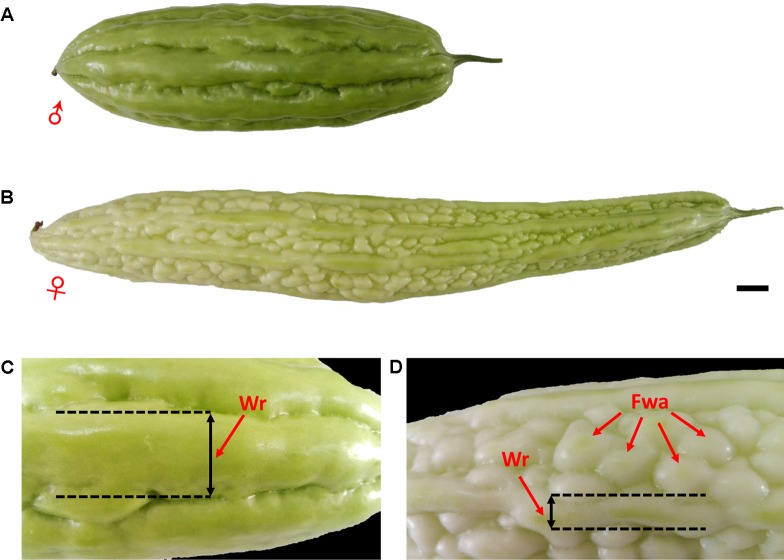
Phenotypic characterization of bitter gourd fruits. **(A,C)** Male parent inbred line ‘Dali-11;’ **(B,D)** female parent inbred line ‘K44;’ scale bar represents 1 cm.

Two cultivated inbred lines of *M. charantia* were used as parents to produce the intra-specific F_2_ mapping population. The female parent, ‘K44,’ is a gynoecious line, with white fruit color, dense round warty epidermis, and broken ridges; the male parent, ‘Dali-11,’ is a monoecious line, with green fruit color, non-warty epidermis, and continuous ridges (**Figure 1**). A total of 423 F_2_ individuals were obtained from crosses of the two parental lines. The whole population was divided into three subsets: 205, 189, and 29 individuals, which were then grown in three different environments: Haikou (N 20.05°, E 110.20°) in spring 2014 (HKS), Guangzhou (N 23.13°, E 113.26°) in spring 2014 (GZS), and Guangzhou in autumn 2014 (GZA), respectively. In the present study, the horticultural traits of bitter gourd investigated included sex expression (gynoecy, gy; first female flower node, fffn; and female flower number, ffn), fruit epidermal structure (fruit wart, fwa; and width of ridge, wr), and immature fruit color (white fruit color, w; and two color measurements: lightness variable, L; and hue angle, H°, measured with a Minolta CR 300 Chroma Meter). In addition to gy, which was investigated in Haikou in spring 2014 (HKS) and in Guangzhou in autumn 2014 (GZA), and ffn, which was investigated in Haikou in spring 2014, the other traits were investigated in Haikou in spring 2014 and in Guangzhou in spring 2014 (GZS). The descriptions of each trait evaluated are shown in **Table 1**.

**Table 1:** Traits evaluated in the present study.

Traits investigated	Description
**Sex expression**	
gy	1: gynoecy; 2: monoecy
fffn	The node number from the first symmetrical true leaves to the first female flower
ffn	The number of female flowers within 30 nodes
**Fruit epidermal structure**	
fwa	1: non-warty epidermis; 2: warty epidermis
wr	Width of fruit ridge measured at the widest place
**Immature fruit color**	
w	1: white fruit color; 2: green fruit color
H°	Calculated from two Minolta CR 300 readings, a and b, *H*° = arctan b/a + 180° (*a* < 0 in this study)
L	Recorded directly from Minolta CR 300 readings

### RAD Sequencing and SNP Identification

Following the protocol of a barcoding system for sample multiplexing, genomic DNA from ‘K44’ and 423 F_2_ individuals were used to build RAD libraries (Baird et al., 2008). Briefly, each sample was digested with *EcoR*I (restriction enzyme cut site is 5′ G^∧^AATTC 3′). This P1 adapter was ligated to the fragments. Then, the ligation products were pooled and randomly sheared with a Bioruptor (Diagenode, Belgium), and DNA fragments with a length of 350–550 bp were gel purified. The P2 adapter, a divergent “Y” adapter, was ligated to the chosen DNA fragments, and the ligation products were purified. The purified fragments were used for PCR amplification, products were gel purified again, and then DNA fragments of 350–550 bp were isolated. The prepared RAD libraries were sequenced on an Illumina Hiseq2000 platform at BGI-Shenzhen (Shenzhen, Guangdong, China) with both paired-end and single-end reads.

After performing standard quality control methods, including removal of the adapters, high missing reads (*N* > 5%), and low-quality reads, a clean data set was obtained and evaluated using the Q20 assessment. All of the clean reads were aligned against the Dali-11 reference genome (scaffold version, unpublished) using SOAP2 (Li et al., 2009). With the deep sequencing of the two parents (‘K44’ and ‘Dali-11’), more stringent quality control criteria, including sequencing depth of the site ≥10×, quality score of consensus genotype >20, rank sum test *p*-value < 1e-5, and average copy number of nearby region <1.1, were applied to identify the genotype of the two parents, finally generating their separated sites. Then, individuals from the F_2_ population were genotyped at the separated sites of the two parents. To ensure the accuracy of genotyping, low coverage sequencing (<7× per site per individual) and low quality (quality score of consensus genotype Q < 20 per site per individual) sites were treated as missing information. After assigning accurate genotypes to F_2_ individuals at the separated sites of the two parents, the genotypes of the individuals were recorded as ‘a’ and ‘b’ when it was the same as ‘K44’ and ‘Dali-11,’ respectively. Heterozygotes were recorded as ‘h’ and missing genotypes were recorded as ‘-.’ The final joined set of SNP genotyping was prepared to construct the genetic map.

### Genetic Map Construction and Scaffold Anchoring

After filtering missing and heterozygous SNPs between two parental lines, and those SNPs with serious segregation distortion (*p* < 0.001, χ^2^ > 13.82), the remaining SNPs were retained for genetic linkage analyses using JoinMap 4.0 (Van Ooijen, 2006). Then, an intercrossed filtering strategy was conducted between individuals and markers based on their missing rate. The logarithm of odds (LOD) threshold was set from three to 15. Finally, high-quality SNPs were split into different linkage groups that were estimated using the Kosambi mapping function. This procedure builds a map by adding loci one by one, starting from the most informative pair of loci, and continuing until all loci have been handled once. The first map node (Map 1), resulting from the first round, is perfect when measured by goodness of fit. Therefore, to further ensure the confidence of linkage groups, the resultant Map1 groups were adopted in this study. Scaffolds were assigned to linkage groups according to their linkage positions and the scaffold could be oriented when more than one marker existed.

### QTL Detection

All traits investigated were used for QTL analysis; phenotypic data for each trait in different environments were analyzed separately. QTL analysis was performed using MapQTL 6 (Ooijen, 2009) software with the powerful MQM mapping (=composite interval mapping) method. Threshold values were calculated using 1,000 permutations and QTLs were designated when genome-wide LOD scores showed the presence of a significant peak at a level of *p* < 0.05. The QTL supported interval was identified by using a 1 LOD unit drop from the peak marker (if no marker exists, the nearest marker was selected). When the same QTL was detected in different environments, the overlapped region was considered the confidence interval for this QTL, and both the genetic interval and physical interval were delimited. Similarly, QTLs were merged if they did not overlap, but were close. The proportion of phenotypic variation explained by each QTL was estimated using the *R*^2^ (%) value of the nearest markers.

## Results

### SNP Development From RAD Sequencing

We generated 193.72 Gb of clean data in total (∼22.28 billion reads) for 423 F_2_ individuals and the female parent ‘K44’ (**Supplementary Table S1**) after using *EcoRI* to digest genomic DNA. The average genome coverage was approximately 18.00%, with an average GC content of 36.92%. The average Q20 of these samples was 98.60% (minimum 97.93%), indicating the high quality of the RAD data (**Supplementary Table S1**). Of these 22.28 billion reads, 93.00% were mapped to the reference genome and used to call SNPs. The number of called SNPs for each sample is presented in **Supplementary Table S2**. The resulting SNPs were filtered and formed a joined set containing 11,016 SNPs.

### Genetic Map for Anchoring Scaffolds

**FIGURE 2:**
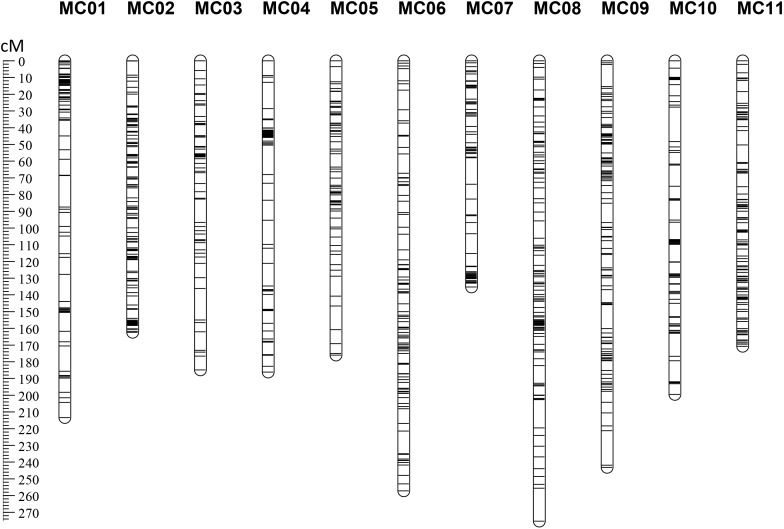
The RAD-based genetic map of bitter gourd for ‘K44’ × ‘Dali-11.’

After removing distorted loci and using an intercrossed filtering strategy between individuals and markers, 249 F_2_ individuals (missing rate <35%) genotyped with 1,512 SNP markers (missing rate <50%) were included in downstream analyses. These SNP loci were assigned into 11 group nodes using JoinMap 4.0 at a LOD threshold of eight to 11, which correspond to the 11 chromosomes of bitter gourd. Then, we performed the map calculations, and the group node had a mapping node and two or three map nodes as child and grandchild nodes, respectively. Eleven Map 1 groups (first map node) containing 1,009 markers (**Supplementary Table S3**) were selected to represent 11 genetic linkage groups (MC01–MC11; **Figure 2**). This map spanned a total of 2,203.95 cM with a mean marker interval of 2.18 cM. Subsequently, a total of 113 scaffolds comprising 251.32 Mb (85.48% of the 294.01 Mb assembled genome) were anchored to the 11 linkage maps and 80 (∼70.80%) of the anchored scaffolds were oriented (**Table 2**). Based on this anchored assembly, the recombination rate of LGs ranged from 6.91 cM/Mb (MC04) to 11.04 cM/Mb (MC10), with an overall value of 8.77 cM/Mb.

**Table 2:** Anchoring of assembled scaffolds to the bitter gourd genetic map.

LG	Number of markers	Genetic distance (cM)	Density (cM/marker)	Number of anchored scaffolds	Number of oriented scaffolds	Physical distance (Mb)	Recombination rate (cM/Mb)
MC01	88	213.39	2.42	8	6	21.14	10.09
MC02	152	162.41	1.07	11	5	21.14	7.68
MC03	61	184.85	3.03	6	5	19.35	9.55
MC04	64	186.19	2.91	13	9	26.96	6.91
MC05	67	175.98	2.63	7	6	19.88	8.85
MC06	103	257.19	2.50	12	9	30.00	8.57
MC07	72	135.26	1.88	11	8	16.38	8.26
MC08	115	275.34	2.39	15	10	34.59	7.96
MC09	109	243.14	2.23	10	7	23.89	10.18
MC10	70	199.52	2.85	10	7	18.08	11.04
MC11	108	170.66	1.58	10	8	19.91	8.57
Total	1,009	2,203.95	2.18	113	80	251.32	8.77

### Inheritance of Traits Investigated

**FIGURE 3:**
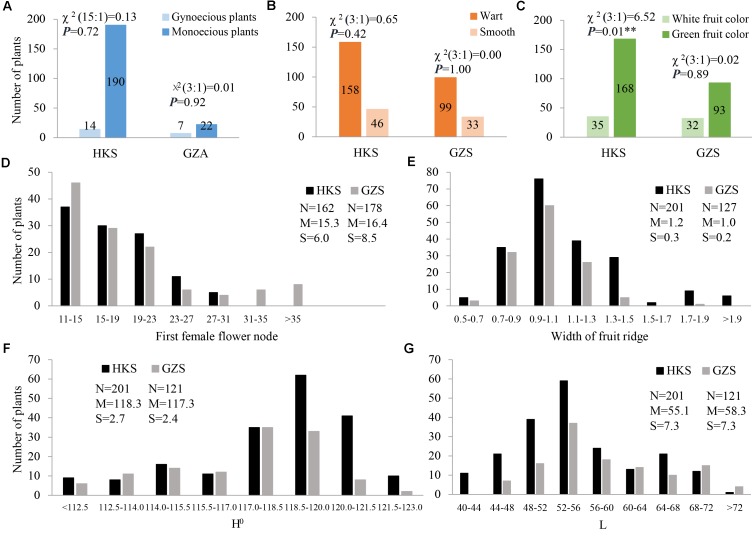
Distribution of phenotypic data for the seven characters investigated in this study. **(A)** gynoecy; **(B)** fruit wart; **(C)** fruit color; **(D)** first female flower node; **(E)** width of fruit ridge; **(F)** hue angle (H°); **(G)** lightness variable (L). HKS indicates the plants were grown in Haikou in spring 2014; GZS indicates the plants were grown in Guangzhou in spring 2014; and GZA indicates the plants were grown in Guangzhou in autumn 2014. N, the number of investigated plants; M, mean; S, standard deviation.

In the present study, three horticulturally important traits involved in sex expression (gynoecy, gy; first female flower node, fffn; and female flower number, ffn), fruit epidermal structure (fruit wart, fwa; and width of ridge, wr), and immature fruit color (white fruit color, w; lightness variable, L; and hue angle, H°) were investigated. The three characters of sex expression showed positive or negative correlations with each other in HKS, but gy and fffn investigated in GZS had no correlation with each other. In particular, the two fruit epidermal structure characters are highly correlated with each other, as are the three immature fruit color characters (**Supplementary Tables S4**, **S5**). The inheritance of these characters was also evaluated. Our data showed that gy, fwa, and w were qualitative characters. Interestingly, the F_2_ plants exhibited monogenic segregation (3:1) for gy in GZA (χ^2^ = 0.01), whereas two-genic segregation (15:1) was identified in HKS (χ^2^ = 0.13). In addition, the F_2_ plants showed monogenic segregation (3:1) for fwa in both HKS (χ^2^ = 0.65) and GZS (χ^2^ = 0.00) and monogenic segregation (3:1) for w in GZS (χ^2^ = 0.02), but distorted segregation in HKS (χ^2^ = 6.52; **Figures 3A–C**). Among the characters investigated, fffn, ffn, wr, H°, and L were determined to be quantitative characters, and fffn was more affected by the environment in which it was grown (**Figures 3D–G** and **Supplementary Figure S1**).

### QTL Analysis

#### Sex Expression

**FIGURE 4:**
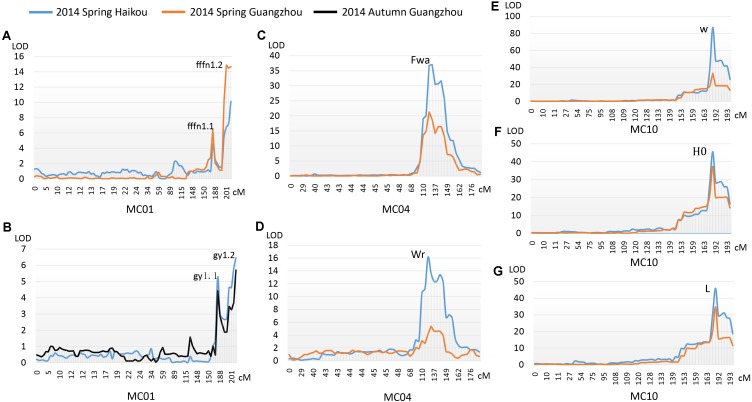
QTLs detected for three horticultural traits. **(A)** The first female flower node (*fffn*); **(B)** gynoecy (*gy*); **(C)** fruit wart (*Fwa*); **(D)** width of fruit ridge (*Wr*); **(E)** white fruit color (*w*); **(F)** hue angle (*H*°); **(G)** lightness variable (*L*).

Two major QTLs were identified on MC01 when bitter gourd was grown in repeat environments for gy, fffn, and ffn: *gy1.1* and *gy1.2*; *fffn1.1* and *fffn1.2*; and *ffn1.1* and *ffn1.2*, respectively. We found that *gy1.1*, *fffn1.1*, and *ffn1.1* were located in the same confidence interval, spanning a genetic interval and a physical interval of 17.74 cM and 976.53 Kb, respectively. Near this region, with an interval of ∼10 cM (∼712 Kb), *fffn1.2*, *gy1.2*, and *ffn1.2* were detected, spanning 15.18 cM and 1,836.22 Kb for genetic and physical intervals, respectively. Therefore, we merged these two regions as the confidence interval of *gy*/*fffn*/*ffn*, which is responsible for sex expression, spanning 42.94 cM and 3,524.92 Kb for genetic and physical intervals, respectively (**Figures 4A,B**, **Table 3** and **Supplementary Figure S2** and **Supplementary Table S6**). After mapping the flanking RAD tags to the OHB3-1 reference, 602 predicted genes were found in this region (**Supplementary Table S9**).

**Table 3:** QTLs detected in ‘K44’ × ‘Dali-11’ F_2_ population of bitter gourd.

Trait	QTL	LG	Environment	Marker interval	Position (cM)	LOD	*R*^2^ (%)	Genetic interval (cM)	Physical interval (Kb)
**Sex expression**									
gy	*gy1.1*	MC01	HKS	scaffold44_3793313- scaffold44_4769840	170.46–188.19	5.27	11.20	42.94 ^b^	3,524.92^b^
			GZA	scaffold44_3793313- scaffold44_4769840	170.46–188.19	4.43	50.50		
	*gy1.2*	MC01	HKS	scaffold44_6022677- scaffold44_7318231	204.30–213.39	6.46	13.60		
			GZA	scaffold44_6022677- scaffold44_7318231	204.30–213.39	5.71	59.60		
fffn	*fffn1.1*	MC01	HKS	scaffold44_3793313- scaffold44_4769840	170.46–188.19	4.50	12.00		
			GZS	scaffold44_3793313- scaffold44_4769840	170.46–188.19	6.43	15.30		
	*fffn1.2*	MC01	HKS	scaffold44_6022677- scaffold44_7318231	204.30–213.39	10.14	25.00		
			GZS	scaffold44_5482008- scaffold44_6022677	198.22–204.30	14.88	32.00		
ffn	*ffn1.1*	MC01	HKS	scaffold44_3793313- scaffold44_4769840	170.46–188.19	7.99	21.20		
	*ffn1.2*	MC01	HKS	scaffold44_6022677- scaffold44_7318231	204.30–213.39	25.12	52.80		
**Fruit epidermal structure**									
fwa	*Fwa*	MC04	HKS	scaffold62_5694078- scaffold62_7255003	121.10–136.78	37.07	56.70	13.64^a^	1,469.87^a^
			GZS	scaffold62_5001233- scaffold62_7163952	112.13–134.73	21.34	52.50		
wr	*Wr*	MC04	HKS	scaffold62_5001233- scaffold62_7163952	112.13–134.73	16.13	30.90		
			GZS	scaffold62_5694078- scaffold62_7255003	121.10–136.78	5.39	17.70		
**Immature fruit color**									
w	*w*	MC10	HKS	scaffold187_352865- scaffold156_898142	176.68–191.92	86.96	86.10	15.25^a^	936.70^a^
			GZS	scaffold187_352865- scaffold156_898142	176.68–191.92	33.11	70.50		
H°	*H°*	MC10	HKS	scaffold187_352865- scaffold156_898142	176.68–191.92	45.61	66.50		
			GZS	scaffold187_352865- scaffold156_898142	176.68–191.92	37.40	75.90		
L	*L*	MC10	HKS	scaffold187_352865- scaffold156_898142	176.68–191.92	46.01	65.20		
			GZS	scaffold187_352865- scaffold156_898142	176.68–191.92	34.77	73.40		

*HKS, GZS, and GZA indicate that the plants were grown in Haikou in spring 2014, Guangzhou in spring 2014, and Guangzhou in autumn 2014, respectively. ^a^overlapped regions; ^b^merged regions.*

#### Fruit **E**pidermal **S**tructure

One QTL was detected on MC04 for each fruit epidermal structure character, *Fwa* and *Wr* for fwa and wr, respectively, explaining 17.70–56.70% of the phenotypic variation in the two environments (HKS and GZS). Both *Fwa* and *Wr* were located in the same confidence interval, indicating they are the same QTL, spanning 13.64 cM and 1,469.87 Kb for genetic and physical intervals, respectively (**Figures 4C,D**, **Table 3** and **Supplementary Table S7**). Then, 218 predicted genes were found in this region (**Supplementary Table S9**).

#### Immature Fruit Color

One QTL (*w*/*L*/*H*°) was detected on MC10 for immature fruit color, which had a higher *R*^2^ value (minimum 65.20%) in the two environments (HKS and GZS). We found that *w*, *L*, and *H°* occupied the same confidence interval, spanning 15.25 cM and 936.70 Kb for genetic and physical intervals, respectively (**Figures 4E–G**, **Table 3** and **Supplementary Table S8**). Accordingly, 128 predicted genes were found in this region (**Supplementary Table S9**).

## Discussion

One of the aims of this research was to construct a bitter gourd genetic map to anchor assembled scaffolds. RAD-seq has been widely used to construct genome wide genetic maps in ryegrass (Pfender et al., 2011), barley (Zhou et al., 2015), peanut (Gupta et al., 2015), apple (Sun et al., 2015), and soybean (Wang W. et al., 2016). In this study, we successfully created a RAD-based genetic map for bitter gourd, comprising 1,009 SNP markers that distributed on 11 linkage groups. The majority of assembled sequences (85.48%) can be anchored to this map, indicating a high genomic coverage of SNP markers. This map provided a basis for integrating assembled scaffolds into a draft genome of bitter gourd, which served as an important step in our ongoing work on sequencing the bitter gourd genome.

This RAD-based genetic map had a mean marker density of 0.46 (maker/cM), which is higher than the 0.30 mean marker density reported by Matsumura et al. (2014) and 0.42 by Urasaki et al. (2017). Nonetheless, it is far from being high resolution. Our study likely recovered a higher mean marker density due to the choice of restriction enzyme (Baird et al., 2008). We used *EcoR*I to digest genomic DNA for RAD-seq analysis, but Matsumura et al. (2014) and Urasaki et al. (2017) used *Pac*I and *Ase*I, respectively. In the future, we recommend the use of additional restriction enzymes when attempting to construct a high density or saturated RAD-based genetic map. Furthermore, the recombination rate is a critical consideration for fine mapping and breeding, and we estimated an overall value of 8.77 cM/Mb for bitter gourd. This recombination rate was significantly higher than 3.28 cM/Mb for cucumber (Huang et al., 2009) and 2.30 cM/Mb for watermelon (Ren et al., 2012), but lower than 13.19 cM/Mb for pumpkin (Zhang et al., 2015). Because a high recombination rate can indicate a large genetic distance over a narrow physical distance, our finding suggests that it is beneficial to use fine mapping methods to determine QTLs/genes for bitter gourd compared with the methods used for species with low recombination rates like cucumber and watermelon.

Another aim of this research was to map genetic loci of three horticulturally important traits involved in sex expression, fruit epidermal structure, and immature fruit color. These traits were evaluated using a combination of qualitative and quantitative data. The repeated QTLs/genes were identified in three environments, and both genetic and physical intervals for each locus were delimited. Currently, we are sequencing a bitter gourd genome dependent on this genetic map, and once the gene annotation is completed, fine mapping of these loci combined with candidate gene analysis will assist in cloning the relevant genes more efficiently.

The inheritance of gynoecy was not completely consistent with previous reports (Ram et al., 2006; Behera et al., 2009; Matsumura et al., 2014); it appeared to be controlled by two pairs of genes in the F_2_ population in Haikou in spring 2014. Both correlation and inheritance analyses indicated that sex expression in bitter gourd is easily affected by the environmental factors in which it is grown, such as the temperature and photoperiod, which can regulate the sex of cucumber flowers (Yamasaki and Fujii, 2003). Matsumura et al. (2014) located the *gy* locus at the distal end of the linkage group, and a similar result was found in this study. The reported SNP marker (GTFL-1) that mapped at a distance of 5.46 cM to *gy* has been aligned at the site of scaffold44_7277273, consistent with our confidence interval (scaffold44_3793313–scaffold44_7318231). Because of its importance, this target region is necessary for further fine mapping. In melon and cucumber, the mechanism of sex determination has been studied in depth. Sexual morphs of melon plants are determined by a combination of alleles at gynoecious (G), andromonoecious (M), and androecious (A) sex loci, controlled by *CmWIP1*, *CmACS7*, and *CmACS11*, respectively (Boualem et al., 2015). Gynoecy in melons is governed by a combination of *Cmwip1/Cmwip1, CmACS7/-, -*/- (the minus symbol indicates any allele at the locus) at the G, M, and A loci, respectively. *CmWIP1* encodes the zinc finger protein WIP1; *CmACS7* and *CmACS11* encode two 1-aminocyclopropane-1-carboxylic acid synthases. Interestingly, no homologs of these genes were annotated in this QTL (*gy*) region. Unlike for cucumber and melon, gibberellin (GA3) is the most effective growth regulator for increasing femaleness for bitter gourd (Ghosh and Basu, 1983). These findings imply that the gynoecy of bitter gourd may be controlled by other genetic mechanisms. Further work should be performed to unveil the genetic determination of sexual morphs in bitter gourd.

Fruit warts and ridges are distinct in shape and epidermal distribution, both of which act as identifying features of bitter gourd. The width of the fruit ridge is correlated with the presence of fruit warts, which provides a novel measurement to dissect the genetic loci of fruit epidermal structure. Our results indicated that the two characters of fruit epidermal structure were possibly controlled by a same dominant gene (*Fwa/Wr*), suggesting a pleiotropic effect. In cucumber, fruit warts consist of spines (non-glandular trichomes) and tubercules governed by *csgl1/mict* (Li et al., 2015; Zhao et al., 2015) and *Tu* (Yang et al., 2014), respectively, and *csgl1/mict* has an epistatic effect on *Tu* (Yang et al., 2014). Recently, *tril*, required for the initiation of trichomes, was reported to show epistasis on the *csgl1/mict* gene (Wang Y.L. et al., 2016). The fruit warts on bitter gourd have no spine, while trichomes can be produced on stems, leaves, fruit stalks, tendrils, and floral organs. Consequently, cloning the genes underlying both fruit warts and trichomes of bitter gourd is essential to understanding the molecular mechanisms that control the formation of fruit warts. Undoubtedly, our results will accelerate the cloning of the *Fwa/Wr* gene.

Immature fruit color is another important trait directly affecting exterior quality. We used data from our observations of fruit color (green and white) and two indirect measurements to delimit the determined gene, *w*, into a narrow physical interval (∼936.70 kb). In cucumber, the *w* gene was confirmed as an *APRR2* (two-component response regulator-like) gene. The white color is caused by a single-base insertion in the *w* gene, which may disable its function in chlorophyll accumulation and chloroplast development (Liu et al., 2015, 2016). Similarly, the white color of bitter gourd fruit is a visible result of a lack of chlorophyll synthesis or disabled development of the chloroplast. The annotation of the bitter gourd genome anchored by the genetic map in the present study will help to directly analyze candidate genes in this physical interval.

## Conclusion

In this paper, we constructed a bitter gourd genetic map using the RAD-seq method. This map successfully assisted in anchoring most of the assembled scaffold sequences. The genetic map and anchored assembly revealed very high recombination rates in bitter gourd. We further used this genetic map and component characters to detect the genetic loci controlling sex expression, fruit epidermal structure, and immature fruit color in bitter gourd. Three reliable QTL/gene loci responsible for these traits were identified through multiple phenotypic data and environments. This study provides the foundation for the assembly of the bitter gourd genome and for further dissecting these horticulture-associated loci.

## Author Contributions

JuC, JiC, and KH conceived and designed the experiments. SL, YN, RH, QW, JS, and NM contributed to field management and conducted the DNA extractions. WH and ZD performed the bioinformatic analyses. JuC wrote the manuscript and KH revised the manuscript. All authors read and approved the final manuscript.

## Conflict of Interest Statement

The authors declare that the research was conducted in the absence of any commercial or financial relationships that could be construed as a potential conflict of interest.

## Supplementary Material

FIGURE S1 Distribution of bitter gourd female flower number investigated in an F_2_ population in Haikou in spring 2014. N, the number of plants; M, mean; S, standard deviation.

FIGURE S2 QTL location of traits involved in sex expression in bitter gourd.

TABLE S1 The mapping rate and coverage of clean reads statistics for each sample.

TABLE S2 The statistics of SNPs detected in each sample.

TABLE S3 List of RAD tags as markers for genetic map construction in this study.

TABLE S4 The correlation coefficient among bitter gourd characters investigated in Haikou in spring 2014 (HKS).

TABLE S5 The correlation coefficient among bitter gourd characters investigated in Guangzhou in spring 2014 (GZS).

TABLE S6 Genome-wide QTL mapping for six expression using multiple phenotypic data and environments.

TABLE S7 Genome-wide QTL mapping for fruit epidermal structure using multiple phenotypic data and environments.

TABLE S8 Genome-wide QTL mapping for immature fruit color using multiple phenotypic data and environments.

TABLE S9 The candidate genes of three genetic loci controlling sex expression, fruit epidermal structure, and immature fruit color in bitter gourd.
