# Supplementary figures and images for: A RAD-Based Genetic Map for Anchoring Scaffold Sequences and Identifying QTLs in Bitter Gourd (Momordica charantia)
Source: Front Plant Sci. 2018 Apr 12;9:477. doi: 10.3389/fpls.2018.00477 (PMC5906717; doi:10.3389/fpls.2018.00477)

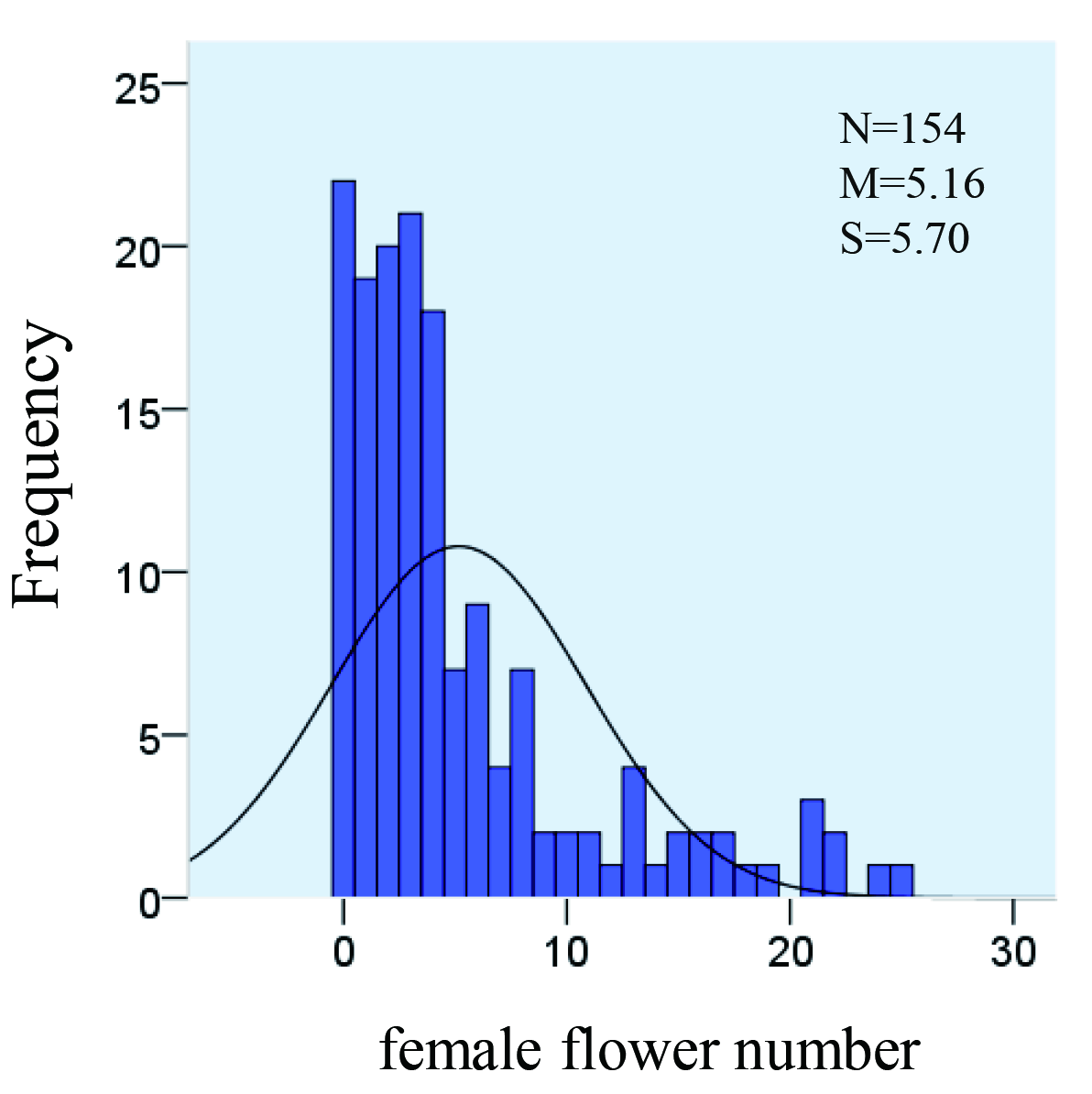

Supplement: FIGURE S1 — Distribution of bitter gourd female flower number investigated in an F2 population in Haikou in spring 2014. N, the number of plants; M, mean; S, standard deviation. [file Image_1.tif]

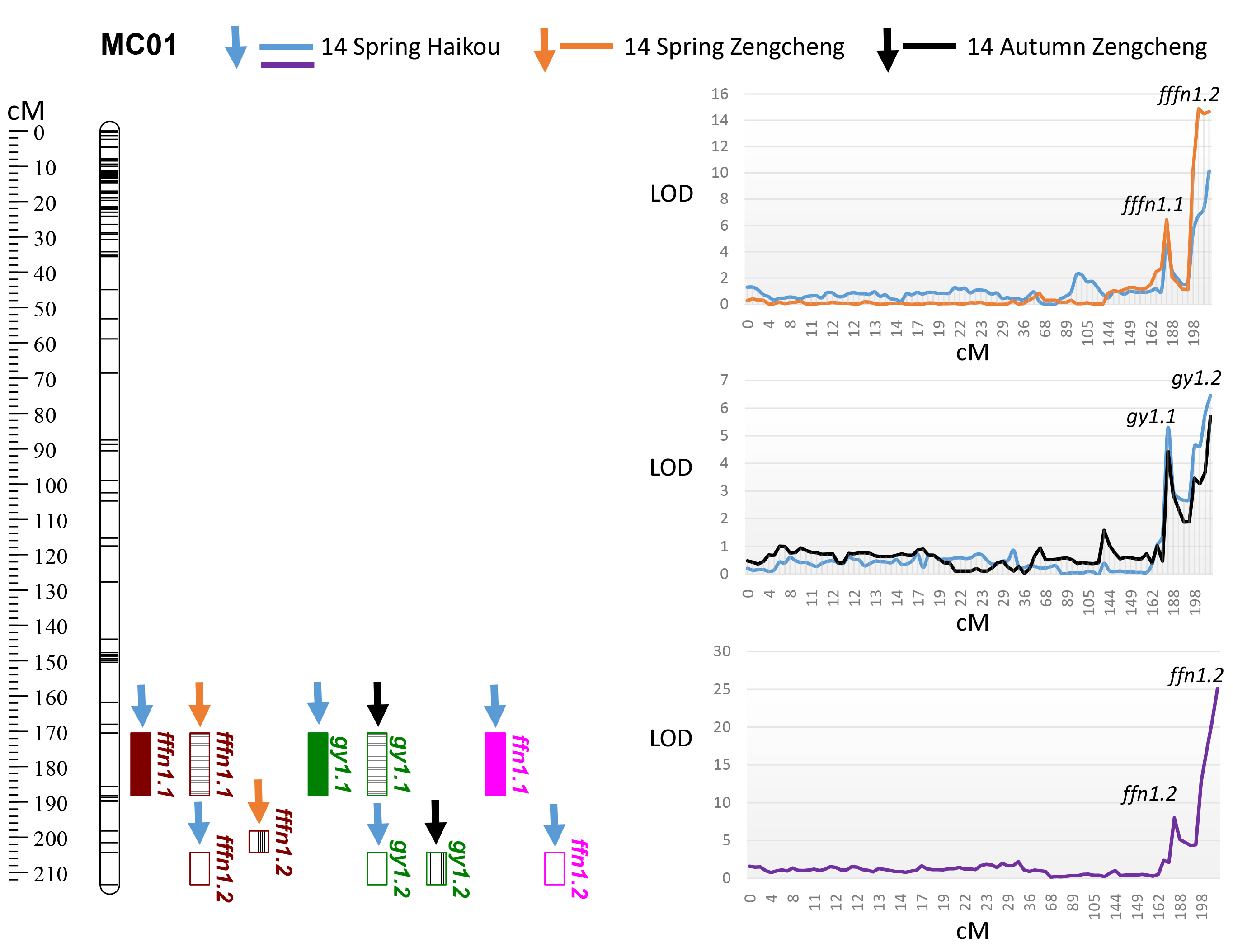

Supplement: FIGURE S2 — QTL location of traits involved in sex expression in bitter gourd. [file Image_2.tif]
